# Correction: Polymerization of C9 enhances bacterial cell envelope damage and killing by membrane attack complex pores

**DOI:** 10.1371/journal.ppat.1010758

**Published:** 2022-08-08

**Authors:** Dennis J. Doorduijn, Dani A. C. Heesterbeek, Maartje Ruyken, Carla J. C. de Haas, Daphne A. C. Stapels, Piet C. Aerts, Suzan H. M. Rooijakkers, Bart W. Bardoel

Fig 2 is incorrect. The x-axis labels for C8 + C9_TMH-1 lock_ and C8 + C9_wt_ + DTT are incorrectly switched in panel B. The authors have provided a corrected version of Fig 2 here.

**Fig 2:**
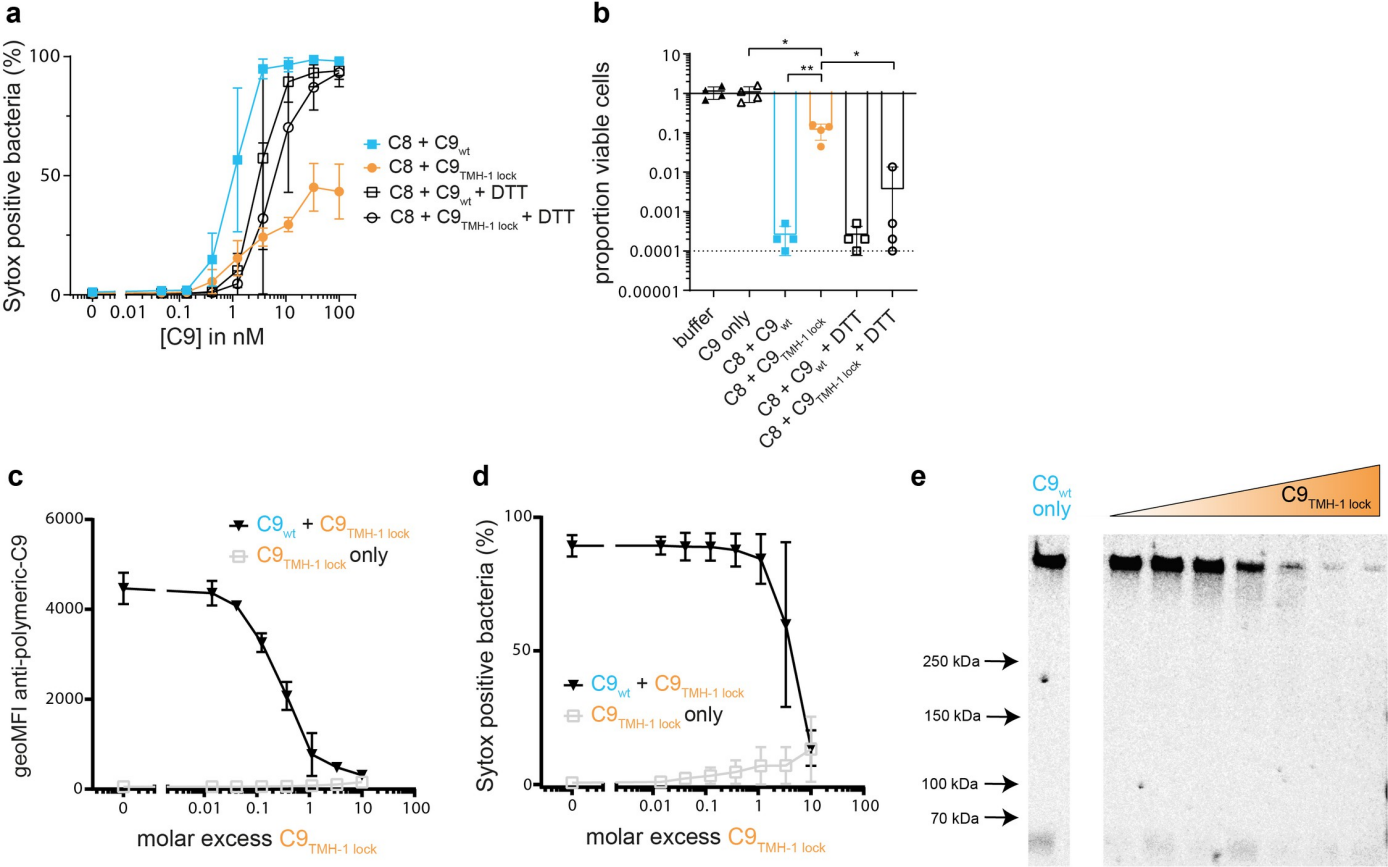
Polymerization of C9 enhances bacterial killing by MAC pores. *E*. *coli* MG1655 was labelled with C5b-7 by incubating in 10% C8-depleted serum for 30 minutes. Bacteria were washed and next incubated with 10 nM C8 for 15 minutes. a-b) A concentration range of C9_wt_ or C9_TMH-1 lock_ was added in the absence or presence of 10 mM DTT for 30 minutes. a) Sytox was used to determine the percentage of cells that have a damaged bacterial IM by flow cytometry as read-out for bacterial killing. b) Bacterial viability was determined by counting colony forming units (CFU’s) and calculating the proportion of viable cells for 100 nM C9 condition (a) compared to C5b-7 labelled bacteria in buffer. The horizontal dotted line represents the detection limit of the assay. c-d) C5b-8 labelled bacteria were incubated for 30 minutes with 20 nM C9_wt_ and a concentration range of C9_TMH-1 lock_. Bacteria were stained with AF488-labelled mouse anti-polymeric-C9 aE11-antibody (c) and Sytox to determine the percentage of cells that has a damaged bacterial IM (d) by flow cytometry. e) Bacterial cell pellets were analyzed by SDS-PAGE for in-gel fluorescence of Cy5-labelled C9_wt_ to distinguish monomeric-C9 from polymeric-C9. From left to right, the molar excess of C9_TMH-1 lock_ from left to right is: 0 (C9_wt_ only), 0.016, 0.05, 0.14, 0.46, 1.1, 3.3 and 10. The SDS-PAGE image is representative for at least three independent experiments. Flow cytometry data are represented by geoMFI values or cell frequencies of the bacterial population. Data represent mean values +/- SD (a, c and d) of three independent experiments or individual values (c) of four independent experiments with mean +/- SD. Statistical analysis was done on ^10^log-transformed data using a paired one-way ANOVA with Tukey’s multiple comparisons’ test (b). Significance was shown as * p ≤ 0.05, ** p ≤ 0.005.
