# Supplementary figures and images for: Polymerization of C9 enhances bacterial cell envelope damage and killing by membrane attack complex pores
Source: PLoS Pathog. 2021 Nov 9;17(11):e1010051. doi: 10.1371/journal.ppat.1010051 (PMC8604303; doi:10.1371/journal.ppat.1010051)

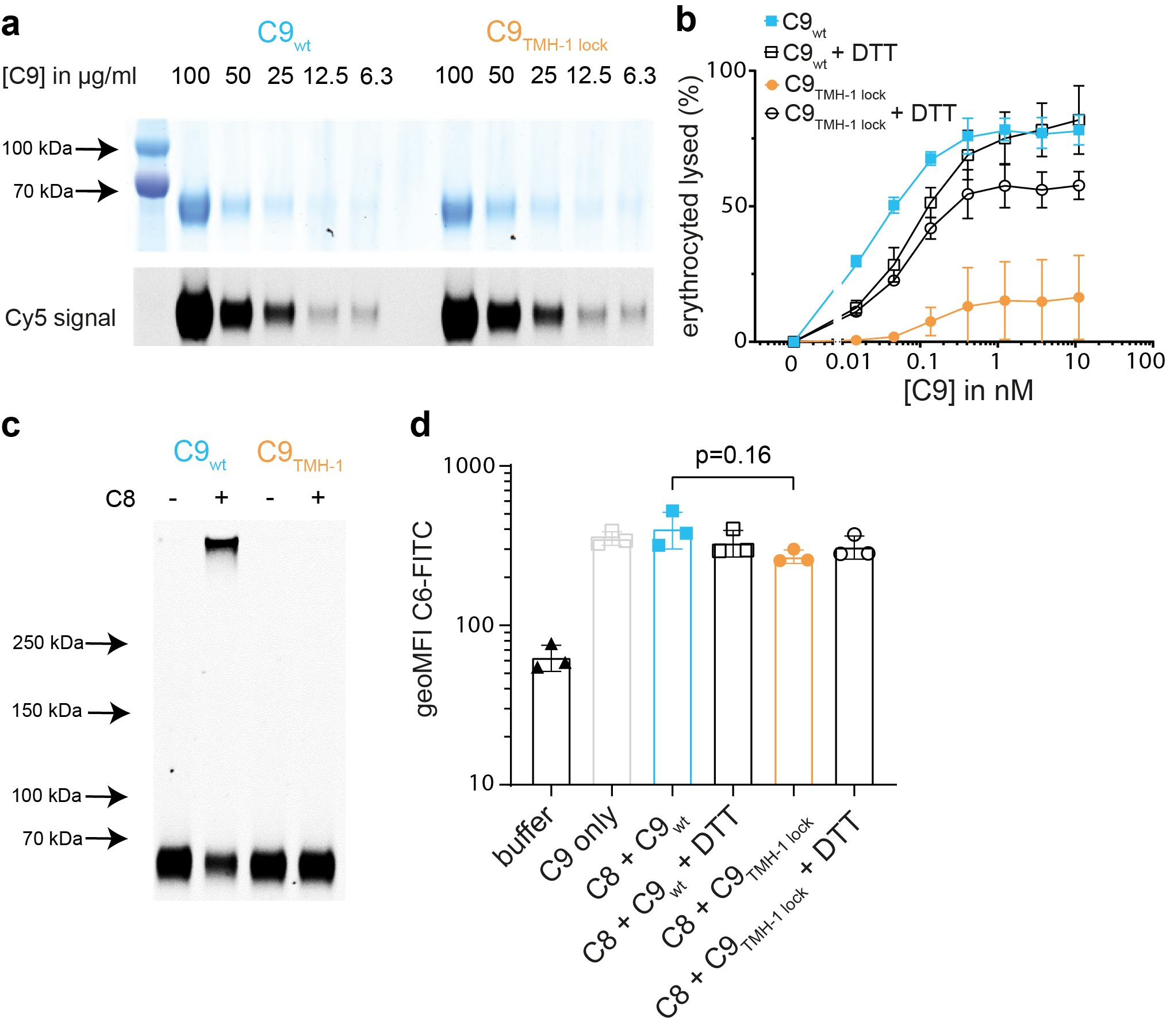

Supplement: S1 Fig — a) SDS-PAGE of a concentration range of Cy5-labelled C9wt and C9TMH-1 lock (in μg/ml). Both InstantBlue staining (top) and in-gel Cy5 fluorescence (bottom) were shown. b) Sheep erythrocytes (4%) labelled with rabbit anti-sheep IgM were incubated in 2% C9-depleted serum for 30 minutes. Next, erythrocytes were washed and incubated with a concentration range of C9wt or C9TMH-1 lock in the presence or absence of 10 mM DTT. The percentage of lysed erythrocytes was calculated by adding MilliQ as 100% lysis or buffer as 0% lysis control after incubation with C9-depleted serum. c) 20 nM pC5b6, 20 nM C7, 20 nM C8 was incubated with 100 nM Cy5-labelled C9wt or C9TMH-1 lock. SDS-PAGE was done to distinguish monomeric-C9 from polymeric-C9 by in-gel Cy5 fluorescence. (d) C6-FITC binding to E. coli MG1655 measured by flow cytometry. Bacteria were labelled with C5b-7 by incubating them in 10% C8-depleted serum supplemented 12 μg/ml C6-FITC with for 30 minutes. Bacteria were washed and next incubated with 10 nM C8 for 15 minutes. Finally, 20 nM of Cy5-labelled C9wt or C9TMH-1 lock was added in the presence or absence of 10 mM DTT for 30 minutes. Flow cytometry data are represented by geoMFI values of the bacterial population. Data represent mean +/- SD (b) or individual values with mean +/- SD (d) of three independent experiments. Statistical analysis was performed using a paired one-way ANOVA with Tukey’s multiple comparisons’ test. For d, data were 10log-transformed. Significance was shown as * p ≤ 0.05, ** p ≤ 0.005, **** ** p ≤ 0.0001. (TIF) [file ppat.1010051.s001.tif]

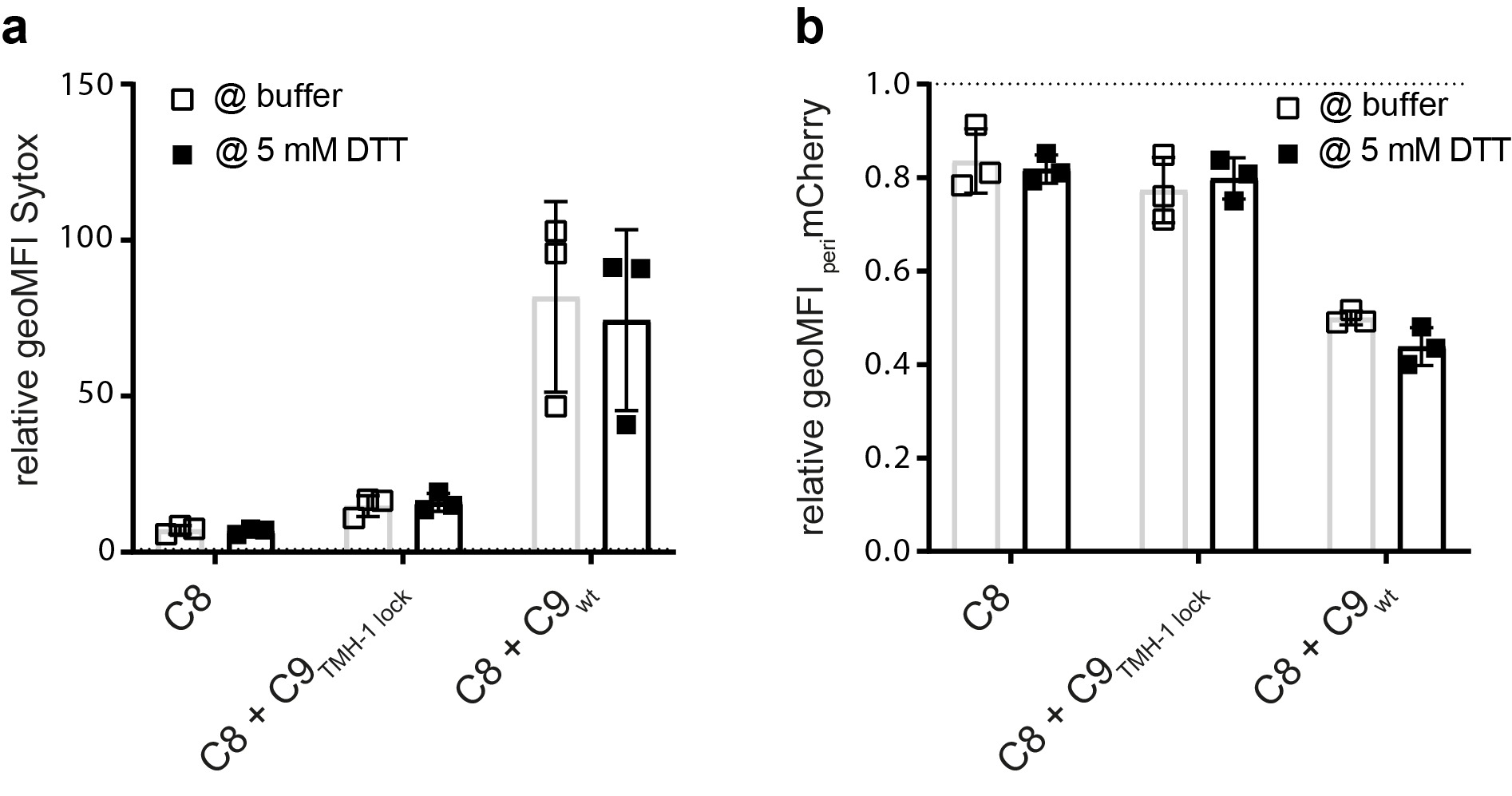

Supplement: S2 Fig — E. coli MG1655 was labelled with C5b-7 by incubating in 10% C8-depleted serum for 30 minutes. Bacteria were washed and next incubated with 10 nM C8 and 50 nM C9wt or C9TMH-1 lock for 15 minutes. Next, bacteria were washed (@) and incubated with buffer or 5 mM DTT for 30 minutes. Bacteria were analyzed by flow cytometry for Sytox influx to determine inner membrane damage (a) and leakage of periplasmic mCherry (perimCherry) for outer membrane damage (b). Flow cytometry data are represented by relative geoMFI values of the bacterial population compared to bacteria in buffer. Data represent individual values with mean +/- SD of three independent experiments. (TIF) [file ppat.1010051.s002.tif]

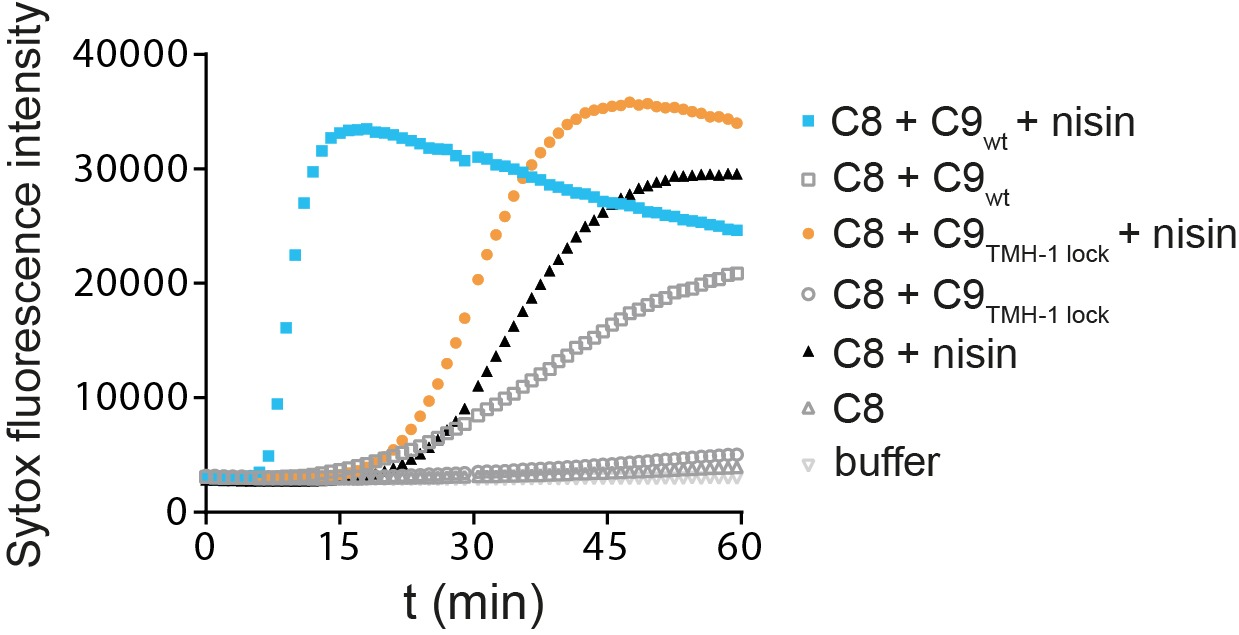

Supplement: S3 Fig — E. coli MG1655 was labelled with C5b-7 by incubating in 10% C8-depleted serum for 30 minutes. Bacteria were washed and next incubated with 10 nM C8 and 20 nM of C9wt or C9TMH-1 lock supplemented with or without 3 μg/ml nisin to measure passage of nisin through the OM. Nisin influx was determined by measuring Sytox influx over time in a multi-well plate-reader assay. The graph shows one representative experiment that has been repeated at least three times. (TIF) [file ppat.1010051.s003.tif]

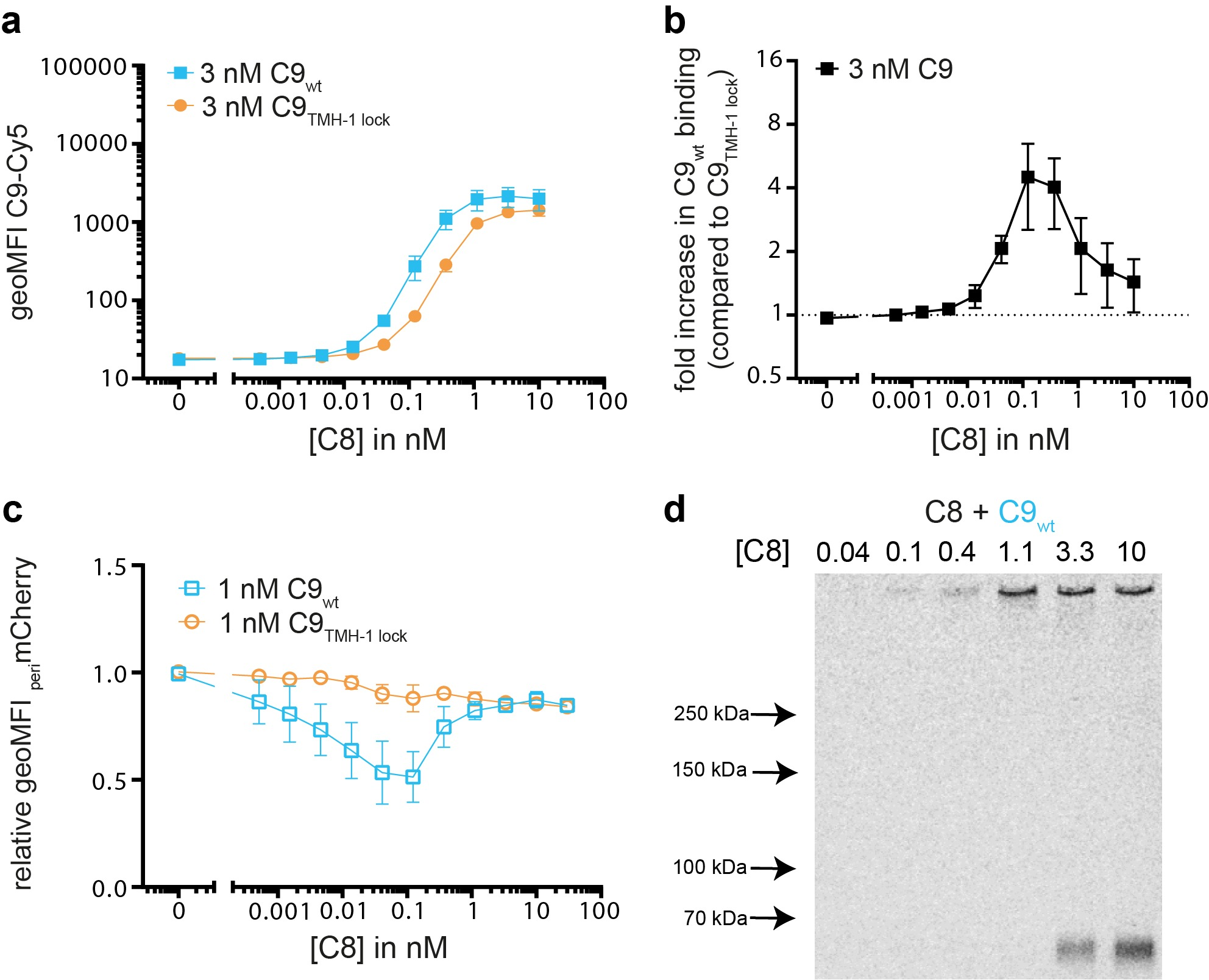

Supplement: S4 Fig — E. coli MG1655 was labelled with C5b-7 by incubating in 10% C8-depleted serum for 30 minutes. Bacteria were washed and next incubated with a concentration range of C8 and 3 nM (a,b,d) or 1 nM (c) Cy5-labelled C9wt or C9TMH-1 lock for 30 minutes. a) Binding of Cy5-labelled C9wt or C9TMH-1 lock to bacteria measured by flow cytometry. b) The relative increase in C9wt binding compared to bacteria labelled with C9TMH-1 lock was calculated as indication for C9 polymerization. c) periplasmic mCherry (perimCherry) leakage was measured after 30 minutes by flow cytometry and represented as relative perimCherry fluorescence compared to t = 0. d) Bacterial cell pellets were analyzed by SDS-PAGE for in-gel fluorescence of Cy5-labelled C9wt to distinguish monomeric-C9 from polymeric-C9. Flow cytometry data are represented by geoMFI values of the bacterial population. Data represent mean +/- SD of three independent experiments. The SDS-PAGE image is representative for at least three independent experiments. (TIF) [file ppat.1010051.s004.tif]

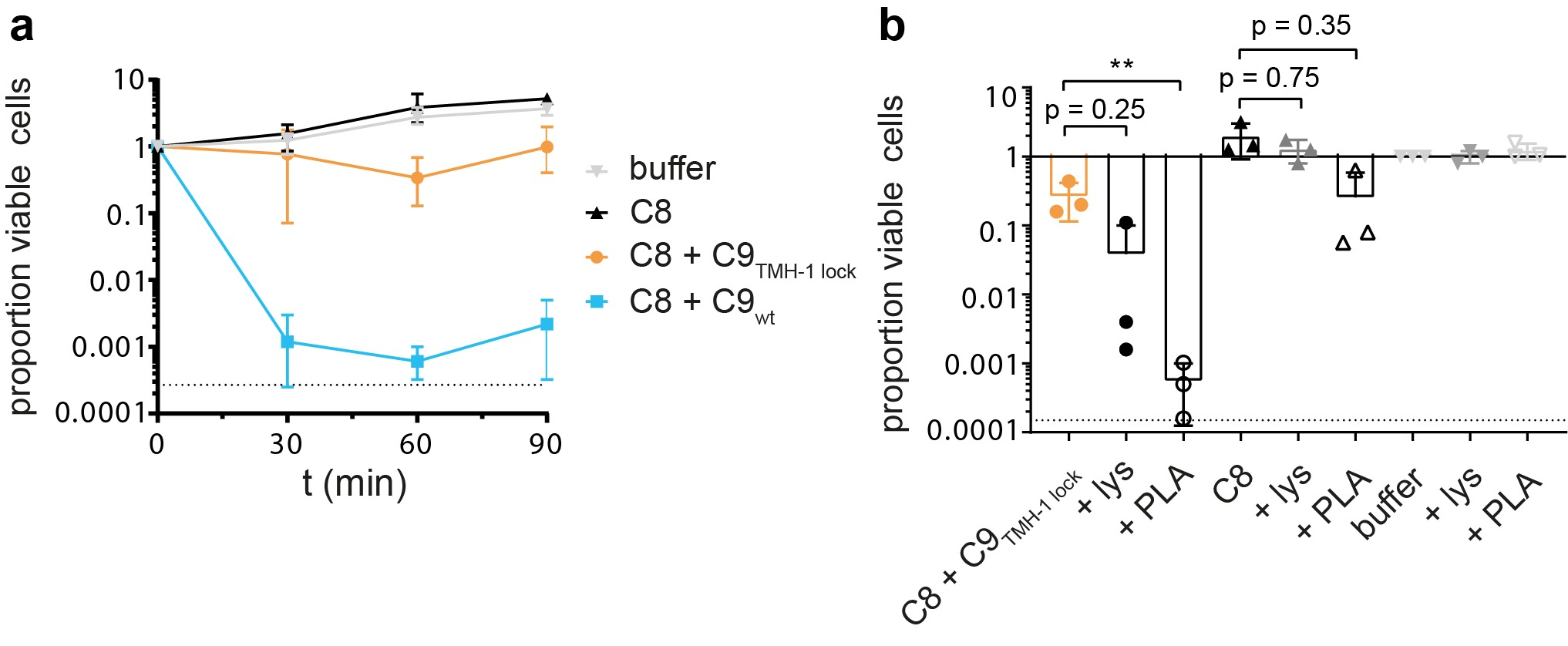

Supplement: S5 Fig — E. coli MG1655 was labelled with C5b-7 by incubating in 10% C8-depleted serum for 30 minutes. Bacteria were washed and next incubated with 10 nM C8 and 20 nM of C9wt or C9TMH-1 lock. a) Bacterial viability was determined at different time points by counting colony forming units (CFU’s) and calculating the proportion of viable cells compared to t = 0. b) E. coli MG1655 bacteria were incubated with 10% C8 depleted serum for 30 minutes. After washing, bacteria were incubated with buffer, 10 nM C8 or 10 nM C8 + 20 nM C9 in the presence of 5 μg/ml lysozyme (lys) or 0.3 μg/ml recombinant type IIa secreted phospholipase 2A (PLA). Bacterial viability was determined after 90 minutes of incubation by counting CFU’s and calculating the proportion of viable cells compared to t = 0. The horizontal dotted line represents the detection limit of the assay. Data represent mean +/- SD (b) or individual values with mean +/- SD (a) of three independent experiments. Statistical analysis was done on 10log-transformed data (b) using a paired one-way ANOVA with Tukey’s multiple comparisons’ test. Significance was shown as ** p ≤ 0.005. (TIF) [file ppat.1010051.s005.tif]

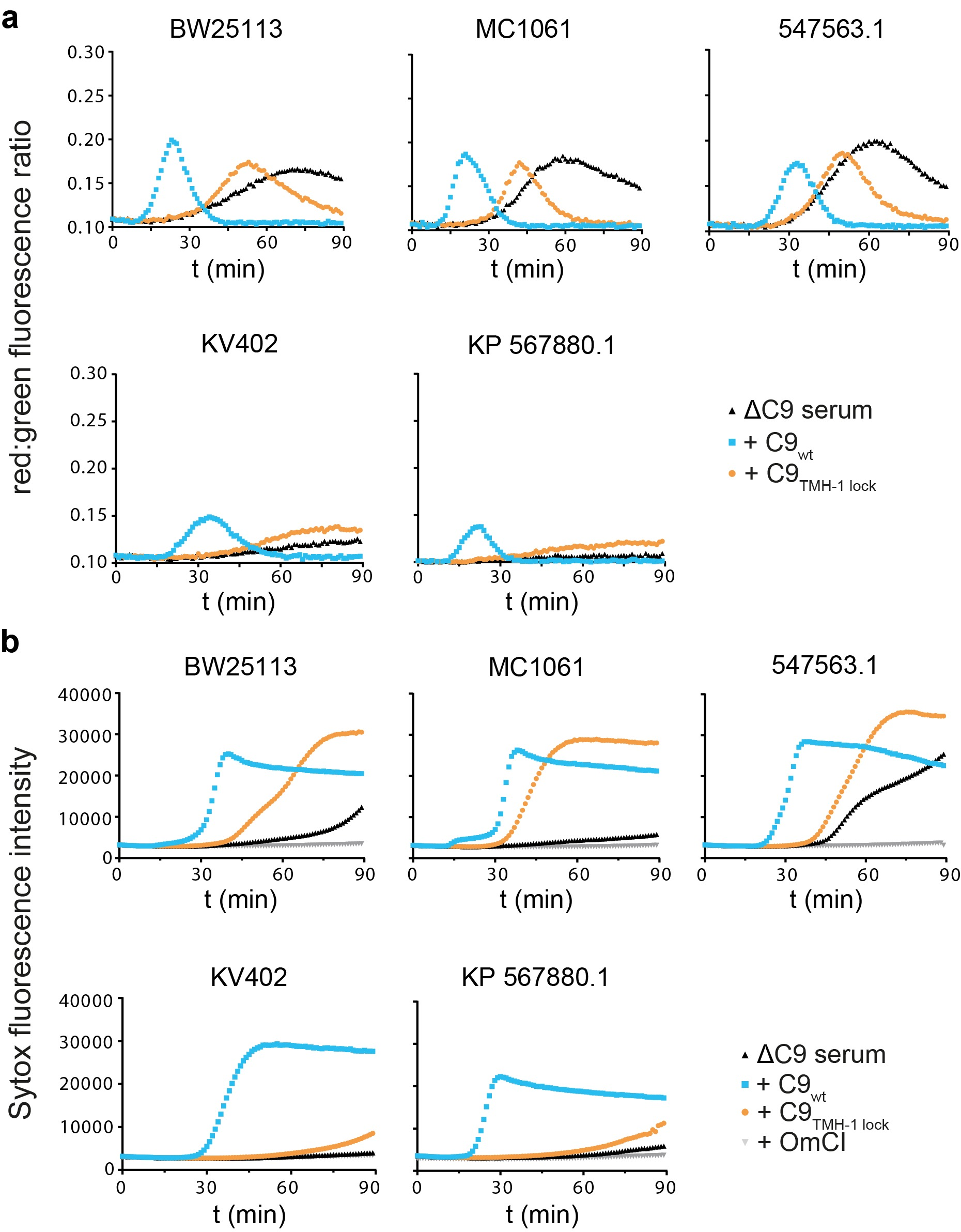

Supplement: S6 Fig — E. coli strains (BW25113, MC1061, 547563.1) and K. variicola 402 were incubated in 3% C9-depleted serum supplemented with a physiological concentration (= 25 nM) of C9wt or C9TMH-1 lock for 90 minutes. K. pneumoniae 567880.1 was incubated in 10% C9-depleted serum supplemented with 80 nM C9wt or C9TMH-1 lock. a) OM damage was measured by DiOC2 influx, which was determined by the shift in red:green fluorescence ratio over time in a multiwell plate-reader assay. b) IM damage was measured by Sytox influx over time in a multiwell plate-reader assay. Graphs represent one representative experiment that has been repeated at least three times. (TIF) [file ppat.1010051.s006.tif]

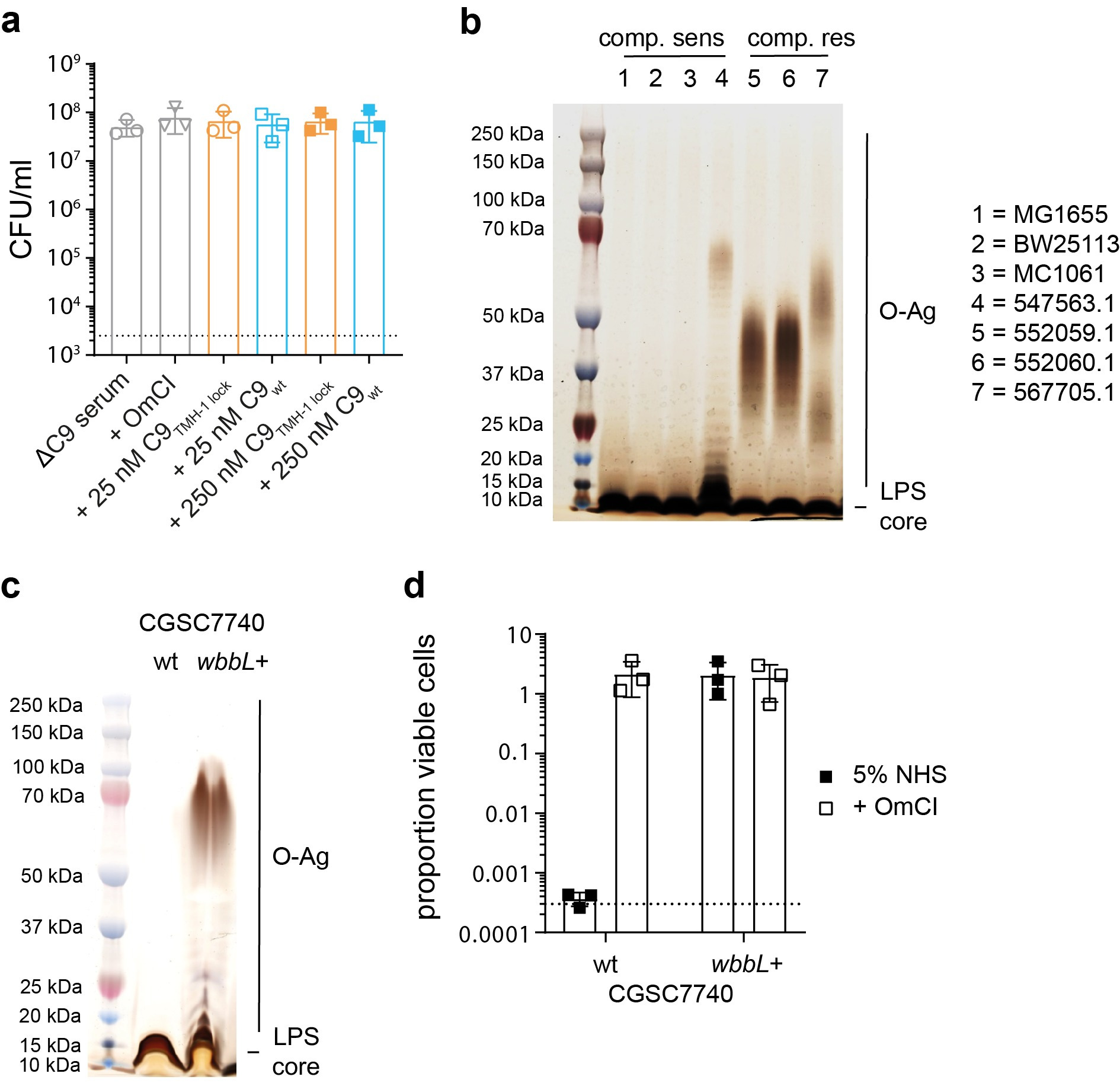

Supplement: S7 Fig — a) Complement-resistant E. coli 552059.1 was incubated in 3% C9-depleted serum supplemented with 25, 75 or 250 nM C9wt, C9TMH-1 lock or 25 μg/ml OmCI. Bacterial viability was determined by counting colony forming units (CFU’s) per ml. The horizontal dotted line represents the detection limit of the assay. b) Complement-sensitive (MG1655, BW25113, MC1061 and 547563.1) and complement-resistant (552059.1, 552060.1, 567705.1) E. coli strains were typed for the presence of LPS O-Antigen (O-Ag) via Silver staining. LPS-core was distinguished from LPS O-Ag based on size. c) CGSC7740 wildtype (wt) and wbbL+ were typed for the presence of LPS O-Ag via Silver staining as done for b. d) CGSC7740 wt and wbbL+ were incubated in 5% normal human serum (NHS) supplemented with and without 25 μg/ml OmCI. Bacterial viability was determined by counting CFU’s and calculating the proportion of viable cells compared to t = 0. The horizontal dotted line represents the detection limit of the assay. SDS-PAGE images are representative for at least two independent experiments. Data represent individual values with mean +/- SD of three independent experiments (b and d). (TIF) [file ppat.1010051.s007.tif]
