# Supplementary material for: Polymerization of C9 enhances bacterial cell envelope damage and killing by membrane attack complex pores
Source: PLoS Pathog. 2021 Nov 9;17(11):e1010051. doi: 10.1371/journal.ppat.1010051 (PMC8604303; doi:10.1371/journal.ppat.1010051)
Supplement: Attachment — Submitted filename: Response letter.docx [file ppat.1010051.s008.docx]

Dear Editor,

We thank the referees for their interest in our study (*Polymerization of C9 enhances bacterial cell envelope damage and killing by membrane attack complex pores*). We are very pleased that the referees consider our findings novel, important and well-designed. Based on the comments given by the referees, we have revised our manuscript in the following manner:

1) We have adjusted some interpretations of our results as suggested by the referees
   a. We have more clearly discussed the possibility of arc-shaped MAC formation (line 318-331)
   b. We have more clearly highlighted the potential residual activity of the C9_TMH-1 lock_ mutant (line 334-346)
   c. We have revised our statements about C9 polymerization being rate-limiting on bacteria (line 195-216 and line 364-378)
2) We have added experimental data (controls/conditions/read-outs) of experiments that were already included in the manuscript that were requested by the reviewers to more strongly support our claims
   a. **Fig. 1f –** aE11 antibody staining of bacteria after DTT treatment
   b. **Fig. 2e -** SDS-PAGE analysis of polymeric-C9 in C9_TMH-1 lock_ competition assay
   c. **Fig. 4e –** SDS-PAGE analysis of C9_TMH-1 lock_ titration on bacteria
   d. **Fig. 4f -** _peri_mCherry leakage of C9_wt_ titration on bacteria
   e. **S1b –** titration of C9_wt_ vs C9_TMH-1 lock_ on erythrocytes
3) We have provided novel experimental data that was not originally included in the manuscript to answers some of the additional questions the reviewers proposed
   a. **S2a, b –** cell envelope damage when C9_TMH-1 lock_ is unlocked after it has bound to C5b-8 on bacteria
   b. **Fig 6d, e +** **S7c, d** – experimental data (LPS Silver staining, bacterial killing and C9 binding by flow cytometry) that directly shows that expression of LPS O-Ag results in resistance to MAC-dependent killing and impaired polymerization of C9 . This has been adjusted accordingly throughout the manuscript (line 22-25, line 38-39, line 86-88, line 283-295, line 384-385 and line 388-396).

In the “point-to-point” response letter below, we respond to the comments raised by the reviewers and explain how we have revised our paper accordingly. All textual changes into the revised manuscript are also highlighted in yellow.

Reviewer #1:

**Part I - Summary**

In this manuscript the authors make use of the ingenious “locked C9” reported by the Dunstone group in 2018 to explore whether MAC-mediated bacterial killing requires C9 polymerisation. The manuscript is very well written and easy to read – for which this reviewer is grateful. I do have some concerns about the presentation and interpretation of the interesting data; these are listed below.

**Part II – Major Issues: Key Experiments Required for Acceptance**

**POINT 1 -** In the abstract and elsewhere the authors state that there has been, prior to the availability of the “locked C9” reagent, “no robust way to specifically prevent polymerization of C9”. I refer the authors to the excellent 1994 paper from McKay and Dankert that uses temperature shifts to do exactly this in the context of bacterial killing. No doubt, the current work builds on this observation but they should fully acknowledge it.

**ANSWER 1 -** We thank the reviewer for pointing out this reference that indeed was currently missing in our introduction and discussion. We have adjusted the text accordingly in our revised manuscript (line 11-13, line 76-77 and line 369-371).

**POINT 2 -** Line 57/58 - At the risk of being pedantic, the description of the MAC in lines 57/58 does not match the description in the references used; in these Bubeck’s group show clearly that the pore is composed of 22 staves, 18 from C9 and one each from C6, C7, C8a and C8b.

**ANSWER 2 -** We have adjusted the text (line 60-62) in the revised manuscript to avoid confusion between the amount of membrane-inserted proteins in the total MAC pore (22) and the total amount of C9 monomers (18).

**POINT 3 -** Similarly pedantic – but important I think; Spicer demonstrated rather than suggested the lock C9 (line 71).

**ANSWER 3 -** We have adjusted the text accordingly (line 78) in the revised manuscript.

**POINT 4 -** Supp1b shows minimal (but not zero) ShE lysis using the locked C9; the Spicer paper found no haemolytic activity in the locked protein and the model proposed would suggest no effect – do the authors think that this is real?

**ANSWER 4 –** Since we consistently observe some residual activity of C9_TMH-1 lock_ on bacteria as well, we think this is a real effect. We have made this more clear in the revised manuscript by adding the full original experiment which included a concentration range of C9_wt_ and C9_TMH-1 lock_ (**S1b**). Spicer *et al*. measured only at one C9 concentration in a different experimental set-up, which makes it possible that they did not pick up this small haemolytic effect of C9_TMH-1 lock_.

**POINT 5 -** Fig 1 - Figures c/d show binding of C9wt and C9lock to C5b-8, replicating what was shown in Spicer et al, albeit in the context of E coli, clarify that this is confirmatory.

**ANSWER 5 -** We have adjusted the text to indicate more clearly what are confirmatory results of the Spicer *et al.* paper and what are new insights (line 78, line 96, line 120-122 and line 307-310). In short, Spicer *et al*. have demonstrated that C9_TMH-1 lock_ can prevent lysis of erythrocytes by C9_wt_, which suggests that it can interfere with polymerization of C9. Our data confirm this in the context of *E. coli*. However, Spicer *et al*. did not yet show direct binding of C9_TMH-1 lock_ to C5b-8. Our data therefore extend on these insights showing direct binding of C9_TMH-1 lock_ to C5b-8.

**POINT 6 -** Figures 1e and S1c show C9 monomer/polymer in slot blots with no markers or other orientations. Here and elsewhere I strongly urge that full gels/blots with clear markers are used.

**ANSWER 6 –** We have adjusted **Fig. 1e**, **Fig. 4d, S1c** and **S4d** accordingly.

**POINT 7 -** Fig 2 - Next, the authors test bacterial lysis using a neat method they have established; Fig 2a shows weak but significant activity in the C9lock – again, given the model proposed, any activity is surprising. Do the authors have an explanation of this? Fig 3c also shows a surprising residual activity in C9lock – state that this “suggests that binding of C9 to C5b-8 in the absence of polymerization slightly increases OM damage”. Again, this doesn’t fit the model – what might the mechanism be?

**ANSWER 7 -** We agree with the reviewer that the residual activity of C9_TMH-1 lock_ is surprising and have discussed this more clearly in our revised manuscript (line 117-119, line 334-346). Nonetheless, our primary goal in this work was to study how polymerization of C9 contributes to bacterial killing, which is why we want to focus our conclusions mainly on the clear enhancement of polymeric-C9 on bacterial killing and cell envelope damage. One explanation for the residual activity of could be that C9_TMH-1 lock_ can still unlock to some extent and form polymeric-C9. Moreover, since C5b-8 is known to already form small lesions in the membrane, binding of C9_TMH-1 lock_ to C5b-8 could enhance the insertion of C5b-8 into the OM.

**POINT 8 -** The impact of releasing the lock on lysis and viability is clear in 2b/c. The dose-dependent inhibition of poly-C9 formation shown in fig 2d is intriguing. Spicer showed very similar data in a different assay system and reached a similar conclusion – that the C9lock could bind and stall polymerisation at different stages; the current data though do beg the question of how many unfolded C9 molecules are needed for the ae11 epitope – given that the mAb detects TCC (2 or 3 C9s) likely not many! Do lower Mr ae11-stained bands appear? Are these sub-pore complexes SDS-labile?

**ANSWER 8 -**  To further clarify if potential intermediate C9 polymers are observed, we have included an SDS-PAGE analysis of bacterial cell pellets of the original competition experiment (**Fig. 2e,** line 160-166) and have discussed these results in line 318-331. This did not indicate the presence of any incomplete C9 polymers. Importantly, this SDS-PAGE analysis does not rely on binding of aE11 because we directly detect the fluorescence of C9-Cy5 in-gel. Although the potential existence of incomplete C9 polymers is indeed an interesting question, we think this is beyond the scope of our manuscript.

**POINT 9 -** The bacterial lysis kinetics in figure 2e look very different – more of a threshold effect and kicking in at equimolar and above – state that this shows “that only very few C9 polymers are required to damage the IM”; however, it could equally show that even complexes with only a few C9s are lytic, the converse of the hypothesis.

**ANSWER 9 –** We agree that the current phrasing does not accurately describe the possibility that incomplete MAC with fewer C9 could also have bactericidal activity and have rephrased this throughout our revised manuscript (line 160-166).

**POINT 10 -** Here and elsewhere I wonder why the authors have not washed after binding C9lock then unlocking in order to properly explore the impact of insertion of that first C9 – seems an obvious addition to many of the experiments that would distinguish effects of C5b-8, C5b-8-C9lock and C5b-8-C9unlock.....Fig3 OM lysis is next explored; 3a/b both show that C5b-8/C9wt causes OM damage while C5b-8/C9lock does not (as expected); a surprising omission is testing the impact of lock release – binding C9lock, washing and unlocking to test impact of C5b-8/C9 (single C9).

**ANSWER 10 -** We have performed the proposed assay and have attached this to our manuscript (**S2a, b)** and have revised the text accordingly **(**line 153-154, line 174-175 and line 850-857). C9_TMH-1 lock_ was bound to C5b-8 on the surface of *E. coli* and afterwards the unbound C9_TMH-1 lock_ was washed away. Unlocking of TMH-1 domain by adding DTT did not increase damage to the IM as measured by Sytox (**S2a**) or passage of _peri_mCherry through the OM (**S2b**), suggesting no effect on cell envelope damage and bacterial killing.

**POINT 11 -** Fig 3c shows a surprising residual activity in C9lock – state that this “suggests that binding of C9 to C5b-8 in the absence of polymerization slightly increases OM damage”. Again, this doesn’t fit the model – what might the mechanism be?

**ANSWER 11 –** As suggested in answer 7, we have discussed possibilities for the residual activity of C9_TMH-1 lock_ more clearly in our revised manuscript (line 334-346).

**POINT 12 -** The data in 4d do not support their statement that SDS-PAGE shows only monomeric C9 at lower concentrations. The authors suggest this indicates that “C9 starts polymerizing when all C5b-8 complexes have bound one C9 molecule”; they note that this interpretation directly contradicts the Parsons study which shows, albeit in an artificial membrane system, that capture of the first C9 is rate-limiting and thereafter further C9 recruitment is rapid. This is an important clarification but I’m not sure that the data fully support it. It seems equally logical to suggest that at low doses all C9 offered is bound, either directly to C5b-8 (only that for C9lock) or both to C5b-8 and C5b-9 (for C9wt); when the C5b-8 sites are saturated the curves diverge, as is seen. Neither poly- nor mono-C9 binding is detected at the low C9 inputs as shown in fig 4d (note comment above regarding slotblots). The strong statement that “these data suggest that polymerization of C9 is rate-limiting in the assembly of complete MAC pores on bacteria” is not well supported by the data – kinetic analyses of the sort described in the Parsons study are likely needed.

**ANSWER 12 –** We agree that our current statements on polymerization being rate-limiting are too strong and have adjusted these in the revised manuscript (line 19-20, line 195-216 and line 364-378). We have also added a new read-out the _peri_mCherry leakage (**Fig. 4f**) that more strongly supports our claim that no C9 polymerizes at low C9 concentrations. This shows that _peri_mCherry starts leaking at the inflexion point of the C9_wt_:C9_TMH-1 lock_ binding ratio (**Fig. 4b**), which coincides with the detection of polymeric-C9 (**Fig 4c, d**). We know this read-out is dependent on C9 polymerization (**Fig. 3a**) and is extremely sensitive, since it happens even with only few MAC pores (Heesterbeek *et al*. EMBO). We therefore still think our data suggest a difference on bacteria versus the data on model-lipid membranes by Parsons *et al*.

**POINT 13 -** Figure 5 shows killing of bacteria in C9-depleted serum supplemented with C9wt of C9lock to explore the requirement for C9 polymerisation for MAC effects on bacteria. These straightforward experiments confirm that polymerisation of C9 in the MAC is essential for efficient bacterial lysis/killing. The findings are complicated by the observation that killing in the presence of C9lock is delayed rather than prevented; they suggest that this is due to continued MAC deposition (supported by OmCI block) and/or presence of other bactericidal serum components. It would be interesting to confirm that there is no “lock-release” in these prolonged serum incubations that might enable polymerisation.

**ANSWER 13 –** As suggested in point 7**,** potential residual polymerization of C9_TMH-1 lock_ has been more specifically mentioned in our discussion of the revised manuscript (line 334-346). However, the main focus of **Fig. 5** and our manuscript is the clear difference in bacterial killing between C9_wt_ and C9_TMH-1 lock_, which suggests bacterial killing is enhanced when C9 can efficiently polymerize. Also, preventing continuous MAC formation by adding OmCI shows this drastically prevents most additional killing in C9-depleted serum with C9_TMH-1 lock_ after 90 minutes (**Fig. 5c**), suggesting any potential residual unlocking of C9_TMH-1 lock_ plays a limited role in bacterial killing in serum.

**Part III – Minor Issues: Editorial and Data Presentation Modifications**

**POINT 14 -** The discussion is clear, although some claims made need to be tempered and some areas suggested as novel more clearly described as confirmations of the Spiller findings – for example, that C9lock binding to C5b-8 is as efficient as WT (already in the Spiller paper which goes way beyond “suggesting”).

**ANSWER 14 –** As discussed in point 5, we have adjusted the text to indicate more clearly what are confirmatory results of the Spicer *et al.* paper and what are new insights (line 78, line 96, line 120-122 and line 307-310).

**POINT 15 -** The suggestion that binding of C9 somehow increases killing capacity of C5b-8 is repeated but how this off-model effect might be explained is not discussed. Although some of the rather strong historical data on MAC killing of bacteria is mentioned (Joiner and Brown – though only a single, albeit important, paper), some highly relevant, particularly from Dankert (see above) and Taylor, data are not included and would strengthen the discussion.

**ANSWER 15 –** We have adjusted the text and have mentioned these papers in our revised manuscript (line 369-371).

Reviewer #2:

**Part I - Summary**

This is a very well written paper that uses innovative tools to evaluate the role of C9 in killing of gram-negatives. This paper unifies several older observations and also lends new insights into the role of C9 in killing gram negatives. Differences across strains and species with respect to how MAC is assembled and inserted have been elucidated. The experiments all have stringent controls. The data have been presented very clearly and the discussion is excellent.

**Part II – Major Issues: Key Experiments Required for Acceptance**

None

**Part III – Minor Issues: Editorial and Data Presentation Modifications**

**POINT 16 -** A minor editorial point – there is no reference to Fig 6d in the text – I think lines 258 and 261 refer to Fig 6d (and not 6c)?

**ANSWER 16 -** We have adjusted the manuscript accordingly and referred to **Fig. 6d (**line 290**)**.

Reviewer #3:

**Part I – Summary**

This work closely follows on from a previous Plos pathogens paper that describes how the MAC facilitates pore formation of the outer membrane of E. coli and delivery of other factors that damage cell wall/membrane integrity. This particular manuscript explores whether the MAC needs to form a full pore or not and how incomplete pore formation affects function although some of the research described as kinetics contradicts published SM, time-resolved work. There is also exploration of how the MAC might be inhibited in complement resistant E. coli. Overall there is an interesting set of experiments but it is difficult to explain as the regain of function of the lock mutant in reducing conditions is not perfect and the DTT does affect the wtC9 function probably due to the partial loss of the normal disulphide bonds required for folding and stability of C9. This data needs to be presented and interpreted in a more upfront manner with the interpretation that the mutant is not completely inactive.

**Part II – Major Issues: Key Experiments Required for Acceptance**

**POINT 17 -** In this sense there is a lot of conceptual overlap with the previous Plos pathogens paper (ref 31 in this manuscript) which shows delivery of other factors. An explanation in the introduction from the authors about then potential overlap and how this is original research would be appreciated. I think that it is really important to put this upfront in the paper as there is a sense that there is redundancy in this manuscript with ref 31.

**ANSWER 17 -** The two studies are different in both aim and concept. We agree that it is good to mention this more specifically, which has been done in line 71-73 and line 313-314. Heesterbeek *et al*. 2021 PLoS Pathogens (now ref 27) describes that MAC requires C9 to permeabilizes the OM for antimicrobial enzymes such lysozyme. However, Heesterbeek *et al*. primarily focus on the functioning of antimicrobial enzymes to bacterial clearance in serum and neutrophils and their dependency on MAC-dependent OM damage in Gram-negative. Here, our aim is to specifically study the role of polymerization of C9 on cell envelope damage. Passage of antimicrobial enzymes through the OM is therefore primarily a tool in our study, but not the research aim.

**POINT 18 -** Figure 1: I find the diagram in figure 1A is confusing as it depicts C9 as binding to C5b-8 and then moving away from C5b-8 as a monomer. The monomer changes shape and then recruits a second monomer of C9. This is not actually how the Membrane attack complex forms and would confuse all readers regardless of whether they are experts or not. This needs to be fixed. The term polyC9 should only be used for the unusual characteristic of human C9 whereby it can form a homomeric ring that looks essentially like a pore but is really a non-physiological phenomenon. This figure needs major work.

**ANSWER 18** – We have adjusted the schematic overview in **Fig. 1a** accordingly to avoid confusion.

**POINT 19 -** Lines 103-5: The author's only use bar graphs to demonstrate the loss of activity for the disulfide trap mutant compared to wtC9. Whilst it might appear to be a clean cut binary style observation that there is either no activity in non-reducing conditions and activity in reducing conditions, when the original paper (Spicer et al.) is looked at, there is a lower LD50 for the reduced disulphide C9 compared to wtC9. Under no circumstances is it acceptable to have only bar graphs of activity for cytolytic protein activity. The original titration based activity profiles (done as replicates) should be presented for this type of data. This will provide a more qualitative view of the activity profile of the proteins being used and is a more honest way of showing the regain of function in the given reducing conditions. Curves should go to a relevantly high concentration of C9 as it is not uncommon for there to be some residual activity. The authors show a loss of wtC9 activity in the presence of DTT but this is not observed in the original Spicer paper so, again, the raw titration based curves would be important here.

**ANSWER 19 -** We have added the full original experiment which included a concentration range of C9_wt_ and C9_TMH-1 lock_ (**S1b**). Spicer *et al*. measured only at one C9 concentration in a different experimental set-up, which makes it possible that they did not pick up this small haemolytic effect of C9_TMH-1 lock_.

**POINT 20 -** Lines 113-123 and Figure 1c, e: I find that the use of the log scale on the Y-axis of Fig 1c dilutes the visual perception of the differences in binding of the trap mutant with and without DTT. In effect the binding of the lock mutant in the DTT is half of wt C9 in DTT. I would say from this data that the C9 lock mutant is half as good at binding/polymerising to the C5b-8 complex and maybe this should be stated as such. This interpretation is also supported by Fig 1e with a much smaller “polyC9” band which was correctly interpreted by the authors.

**ANSWER 20 –**We have more clearly stated that our C9-Cy5 binding data (**Fig. 1d**) reveal that DTT only partially restored polymerizing capacity of C9_TMH1-lock_ by DTT in line 132-133, which corresponds with SDS-PAGE (**Fig. 1e**) and antibody staining (**Fig. 1f**). Data in **Fig. 1c** primarily highlight that C9_TMH-1 lock_ can still bind to C5b-8, which has not yet been shown before. As flow cytometric values cannot reach 0, this is best represented on a logarithmic scale.

**POINT 21 -** What is missing is also the binding of aE11 to the reduced C9 lock mutant in Figure 1f to quantitate the formation of polymerised C9 and a more honest, quantitated statement of the amount of C9 polymerisation in the reduced form of the C9 lock mutant. I.e. it is not a perfect regain of wt-like activity and should be interpreted as such.

**ANSWER 21 –**We agree that this condition would add to our manuscript and have added this condition in **Fig. 1f**. The maE11 staining is in line with the C9_wt_:C9_TMH-1lock_ ratio (**Fig. 1d**) and the polymeric-C9 by SDS-PAGE (**Fig. 1e**).

**POINT 22 -** Figure 2a,b: It would be easier for the reader to interpret the experiments correctly if you combined Figures 2 a and b.

**ANSWER 22 -** We have revised the manuscript accordingly.

**POINT 23 -** Lines 142-144, figure 2d,e: I find this to be a really important observation that could be expanded. Is it possible to interpret that arcs are able to allow the damage of the inner membrane at this stage of the manuscript? And refer to the background of data of arcs which is quite extensive for the MACPF/CDC family.

**ANSWER 23 -** It is indeed possible that arc-shaped MAC pores could damage the bacterial inner membrane and we have discussed this possibility more clearly in our revised manuscript (line 160-166 and line 318-331).

**POINT 24 -** Lines 149 - 55, Figure 3a: the y-axis is not a linear-scale not a log scale. Not consistent with the other experiments. Is this experiment compared to E. coli not expressing peri-mCherry for background scattering as the baseline? Overall this is a weak observation but that is fine. The weak change may suggest that even with wtC9 there are only a few full pores that are capable of passaging mCherry as opposed to the passage of nisin and this should be noted in the results interpretation. Statistics need to be assigned to state it this is a significant observation or not as it looks like it is not. Maybe present the nisin work which is more significant before the mCherry work and state clearly the potential for arc formation as a possible explanation.

**ANSWER 24 –** The y-axis was represented as a linear scale and has been adjusted to a 2log scale, to account for the fact that relative values cannot reach 0. The decrease of _peri_mCherry signal (~50% of the initial signal) as seen in our manuscript by flow cytometry is specific for a the complete loss of mCherry in the periplasm, which has been validated by confocal microscopy (Heesterbeek et al. EMBO 2019). Since C9_wt_ decreases mCherry signal to a maximum extent within 5 minutes, whereas C5b-8 with C9_TMH-1lock_ or C5b-8 alone do not reach maximum decrease within 60 minutes, we think this is in fact a clear observation.

The potential of arc formation has been more extensively discussed in the discussion of our revised manuscript (line 318-331).

**POINT 25 -** Lines 168-72: Given that there is potential background pore formation for the C9 lock mutant which should be explained in a more upfront way (i.e. Figure 1c), then a better/alternative explanation for Fig 3c is that there is still some background MAC formation that allows the passage of DiOC2. Again I think this is perfectly logical. There is also evidence of leaking membranes with the C5b-8 complex which may explain the black triangle curve.

**ANSWER 25 –** We have highlighted the potential of residual C9 polymerization by C9_TMH-1 lock_ at **Fig. 1e** more up front (line 117-119) and have more extensively discussed in the discussion of our revised manuscript (line 334-346) .

**POINT 26 -** Discussion: There definitely needs to be discussion about the role of arcs or incomplete MAC pores that can allow some passage of some molecules (DiOC2 and nisin) but not mCherry. And this should be reflected in the model presented in Figure 7 (just add an arc between step iii and step IV.)

**ANSWER 26 –**As already mentioned at point 23, we have adjusted our manuscript accordingly and have more extensively discussed the potential role of arc-shaped MAC in our discussion (line 318-331). We have also adjusted our figure legend for **Fig. 7** (line 825) to ensure that the possibility of arc-shaped MAC is also included. However, we think that adding an arc-shaped MAC intermediate in **Fig. 7** would be an over-interpretation of our results. Our data do not provide any direct evidence that suggests the existence of arc-shaped MAC and previous AFM analyses of MAC on bacteria have primarily revealed ring-shaped MAC pores in the presence of an excess of C9, and no arc-shaped MAC (Heesterbeek et al. 2019 EMBO, Doorduijn et al. 2020 PLoS Pathogens). If there is arc-shaped MAC, it could therefore be primarily a result of actively limiting the capacity of C9 to polymerize or because of the mutation conferring the lock in the TMH-1 domain. We therefore think that these changes in the text of the revised manuscript sufficiently highlight the possibility of arc-shaped MAC.

**POINT 27 -** Lines 189-90: “Altogether, these data suggest that polymerization of C9 is rate-limiting in the assembly of complete MAC pores on bacteria.” This needs to be more clearly stated that the C9:C9 interaction is slower than the initial C8:C9 interaction. Having said this, it is impossible to reconcile this whole E.coli, ensemble data with published single molecule, time resolved data (AFM work published in ref 30) that provides more reliable evidence of the opposite kinetic observation? With this kind of ensemble data that relies on titration of protein then it isn’t really a true kinetic study.

**ANSWER 27 -** We agree that our current statements on polymerization being rate-limiting are too strong and have adjusted these in the revised manuscript (line 19-20, line 195-216 and line 364-378).

**POINT 28 -** Figure 4d: Would be complete with the Western blot of the C9 lock mutant as well.

**ANSWER 28 –** We have added the SDS-PAGE analysis of C5b-8 labelled bacteria incubated with a titration of C9_TMH-1 lock_ (**Fig. 4e**).

**POINT 29 -** Lines 235 - 262: There is not enough data to support the interpretation of the impaired C9 polymerization on complement resistant E. coli being due to the improper anchoring of C5b-7. Further explanation or experiments would be needed for this section and it seems rather immature.

**ANSWER 29 –** We agree that these data are too preliminary to conclude that and have omitted these data from our revised manuscript. Although not a direct answer to this question, we have provided additional experiments (**Fig. 6d, e + S7c, d**) to the revised manuscript that more directly show that expression of LPS O-Ag in a complement-sensitive O-Ag negative strains results in impaired polymerization of C9 and complement-resistance. We have adjusted the revised manuscript accordingly (line 22-25, line 38-39, line 86-88, line 283-295, line 384-385 and line 388-396).

**Part III – Minor Issues: Editorial and Data Presentation Modifications**

**POINT 30 -** Reviewer #3: Lines 109-10: “These data confirm that the capacity of C9TMH-1 lock to form polymers is impaired, and can be reversed by reducing the cysteine bridge lock.” Figure S1c does not show the “C9TMH1 lock” forming polyC9 in reducing conditions (in SDS-PAGE) and so this statement has not been experimentally qualified. This is later covered by analysis of Fig1e so move to that section?

**ANSWER 30 –**We have adjusted the text accordingly (line 132-133).

**POINT 31 -** Lines 138-9: “Reducing C9TMH-1 lock with DTT restored its capacity to damage the IM (Fig. 2b)” Add at the end of this phrase the following or equivalent “relative to wtC9 in reducing conditions which had overall reduced activity compared to wtC9 in non-reducing conditions”

**ANSWER 31 –** We have adjusted the revised manuscript accordingly (line 151-153).

**POINT 32 -** Figure 2c figure legend line 666: is this 3 or 4 “individual values (c) of three independent experiments with mean +/- SD. Also the SD bar for the far right bar looks truncated?

**ANSWER 32 –** The figure legend has been adjusted accordingly to show that these were 4 individual values (line 749) and truncated error bar has been fixed.

**POINT 33 -** Line 243 and other parts of methods. Make sure that you have appropriate units after each number full stop for example 10% c5-depleted serum should have volume per volume after the 10%

**ANSWER 33 –** This has been adjusted accordingly in the methods section.

**POINT 34 -** Line 456 why was there no tenfold dilution?

**ANSWER 34 –** The ten-fold dilution was not required as the four specified dilutions already allow good quantification of the CFU’s in our experiments.
